# The best way of preventing device–device interactions may be avoiding implanting 2 devices in the first place

**DOI:** 10.1016/j.hrcr.2023.02.018

**Published:** 2023-03-01

**Authors:** Ralph J. Verdino

**Affiliations:** Perelman School of Medicine, University of Pennsylvania, Philadelphia, Pennsylvania

## Abstract

Extensive testing during the second device implantation was required to best program the devices in order to decrease these negative interactions. Unfortunately, these programming changes were not always successful at preventing device–device interactions during clinical follow-up.

Device–device interactions were somewhat common problems a couple of decades ago when only single-chamber defibrillators were available. During those times (and even today) many patients undergoing defibrillator implantation had cardiac conduction disease with or without sinus node dysfunction and already had pacemakers before undergoing implantable cardioverter-defibrillator (ICD) implantation. Other patients with a single-chamber ICD developed bradyarrhythmias owing to aging of the conduction system or to myocardial infarction, or as a result of required medications (such as beta-blockers, non-dihydropyridine calcium blockers, and amiodarone), and ultimately required implantation of a second device, a dual-chamber pacemaker.

Back then, a major concern was how devices interacted with each other. A true episode of ventricular fibrillation could be undersensed by a pacemaker, causing it to pace. The pacing spikes could be seen by the ICD as normal beats and cause the defibrillator to undersense the underlying ventricular fibrillation owing to issues of automatic sensitivity adjustments. This could result in failure to shock ventricular fibrillation and, ultimately, patient death.

Another common device–device interaction was double or even triple counting by the defibrillator of native beats (including paced QRS complexes and large-amplitude T waves caused by ventricular pacing) and the pacing spikes themselves in 1 or both chambers. This often led to multiple inappropriate ICD discharges within a few minutes, causing debilitating anxiety in many patients.

Today, with dual-chamber and cardiac resynchronization defibrillators, a single device is implanted de novo, or an upgrade from a single-chamber ICD is undertaken for patients with bradyarrhythmias who require ICD implantation or ICD patients who require pacing. Device–device interactions were waning—until, that is, the advent of the subcutaneous implantable cardiac defibrillator (S-ICD).

Since pacing in the S-ICD only occurs during postshock bradycardia, patients who already have pacemakers are rarely implanted with S-ICDs; and patients with S-ICDs who develop bradyarrhythmias are often upgraded to a dual-chamber or CRT-defibrillator, and have their S-ICD removed. This is the way we can avoid device–device interactions. Unfortunately, there still are some patients who are left with 2 devices—a pacemaker and a defibrillator—and are at risk for the mayhem caused by device–device interactions.

In the case report “Atrial pacing–induced oversensing in subcutaneous implantable cardioverter-defibrillator” by Wharmby and colleagues,^1^ a case of device–device interaction is described. The patient is an 18-year-old man with hypertrophic cardiomyopathy. He reportedly had a primary prevention transvenous ICD for at least 5 years (likely implanted at or before age 13) that had been removed for infection and changed to an S-ICD. He now presented with conduction disease described as “first-degree atrioventricular (AV) block (200–250 ms) and right bundle branch block (RBBB) (QRS 134 ms) with right axis deviation.” Instead of upgrading his S-ICD to a dual-chamber ICD, a dual-chamber permanent pacemaker was added. The pacemaker was set for an upper tracking rate of 150 beats/min, while the ICD was set to shock for heart rates at or above 250 beats/min. This is a recipe for inappropriate ICD discharges, as upper tracing rates should be set for less than half of the ICD treatment rate to avoid double counting of the pacing spike and the QRS complex. Ideally, setting the upper tracking rate to less than one-third of the ICD shock rate would avoid an unnecessary shock for triple counting. It seems like the implanters who placed the pacemaker (not the authors of this case report) did not test for interactions during or after the addition of a pacemaker to this young man.

Triple counting was noted on device interrogation, and fortunately was interrupted by a spontaneous premature ventricular complex. It did not cause any ICD discharges, and fortunately was recognized by the authors, who removed both devices and implanted a CRT-defibrillator.

This case highlights the frequently quoted tenet in medicine that “more is not always better.” We must think carefully about what we are implanting, and ask, “Is it really necessary?” and “What are the risks and benefits of what we are doing or not doing?” This 18-year-old man has already undergone 4 device implantations—without much of an apparent benefit from any of them thus far. Hopefully, this will be the only case report written about him, in a long life ahead.
